# Surgical Management of Pediatric Venous Malformations Misdiagnosed As Infantile Hemangiomas: A Report of Two Cases

**DOI:** 10.7759/cureus.94663

**Published:** 2025-10-15

**Authors:** Zineb Benmassaoud, Yacine Zouirech, Badr Rouijel, Achraf Miry, Bouchra Mouaouya, Mohamed A Oukhouya, Sanae Abbaoui, Hind Cherrabi

**Affiliations:** 1 Pediatric Surgery, Souss Massa University Hospital Center, Ibn Zohr University, Agadir, MAR; 2 Pediatric Surgery, Children's Hospital, Ibn Sina University Hospital Center, Mohammed V University, Rabat, MAR; 3 Pathology, Souss Massa University Hospital Center, Agadir, MAR; 4 Pathology, Agadir Pathology Center, Agadir, MAR

**Keywords:** infantile hemangioma, issva classification, pediatric surgery, propranolol, vascular anomaly, venous malformation

## Abstract

Venous malformations (VMs) are low-flow vascular malformations that must be distinguished from vascular tumors such as infantile hemangioma (IH). Misclassification leads to inappropriate therapy and delayed definitive management.

We report two children with congenital, enlarging, compressible subcutaneous masses (inframammary and dorsal). Both had been labeled IH in infancy and received oral propranolol with no response. MRI showed lobulated subcutaneous lesions, T2 hyperintense and T1 iso-/hypointense, without arterial flow voids or invasion. Both underwent complete surgical excision; reconstruction used a split-thickness skin graft in case one and primary closure in case two. Histopathology demonstrated dilated, thin-walled venous channels lined by bland endothelium, confirming VM. Recovery was uneventful with satisfactory cosmetic outcomes and no early recurrence.

In compressible, bluish lesions present at birth and growing proportionally, the International Society for the Study of Vascular Anomalies (ISSVA) framework and MRI features should guide diagnosis. Propranolol is ineffective for VMs. For well-circumscribed subcutaneous lesions causing symptoms or psychosocial burden, complete excision is a safe and effective option.

## Introduction

Vascular anomalies represent a heterogeneous group of disorders, and accurate classification is essential for optimal management. The International Society for the Study of Vascular Anomalies (ISSVA) distinguishes vascular tumors, such as infantile hemangiomas (IHs), from vascular malformations, the latter being structural anomalies without endothelial proliferation (e.g., venous malformations) [1,2]. This distinction is critical, as natural history, imaging work-up, treatment, and prognosis differ substantially between these entities [1,2].

IHs are benign vascular tumors characterized by endothelial proliferation with rapid growth during early infancy, followed by spontaneous involution. They typically respond dramatically to oral propranolol, which is now considered first-line therapy [3,4]. In contrast, venous malformations (VMs) are congenital malformations of the venous system, composed of structurally abnormal, non-proliferative vessels. They enlarge proportionally with the child, persist throughout life, and neither respond to β-blockers nor undergo spontaneous regression [5,6]. On MRI, VMs are classically T2-hyperintense and T1 iso-/hypointense, often lobulated, lacking arterial flow voids, and frequently containing phleboliths. MRI is essential to delineate their extent and confirm low-flow behavior [5-7].

Because of overlapping clinical features, VMs are frequently misdiagnosed as IHs in early childhood, leading to delays in definitive care and unnecessary exposure to ineffective therapies such as propranolol [1-4]. Advanced imaging, particularly MRI together with histopathology, is crucial for accurate diagnosis and treatment planning, as it confirms low-flow physiology and excludes alternative entities [2,5-7].

We report two pediatric cases of subcutaneous VMs initially misclassified as IHs and treated unsuccessfully with propranolol; surgical excision subsequently confirmed the diagnosis in both cases. These observations underscore the importance of strict adherence to ISSVA classification, highlight the central role of appropriate imaging and histopathology, and demonstrate the value of surgery for selected, well-circumscribed symptomatic lesions.

## Case presentation

Case one

**Figure 1:**
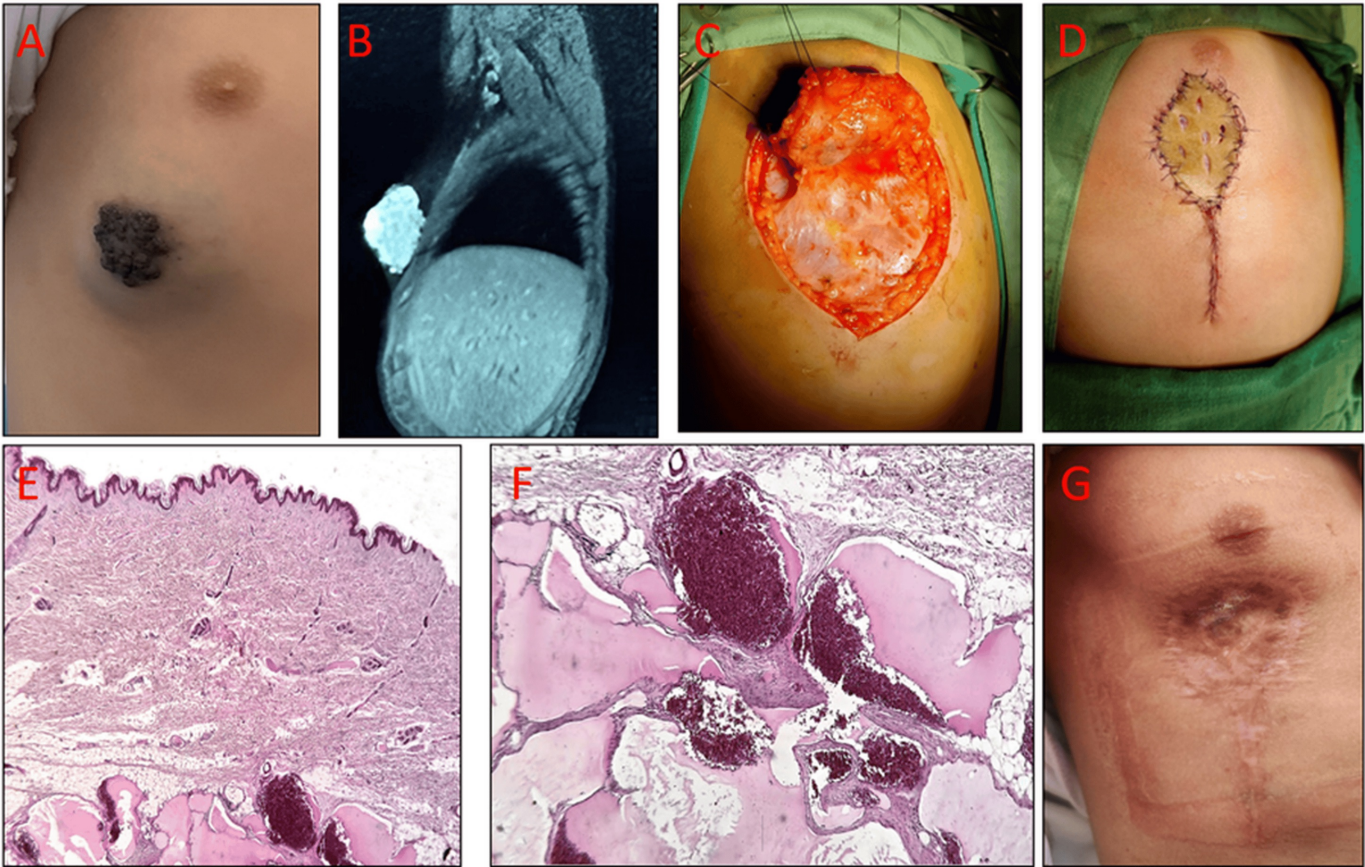
Right inframammary venous malformation (case one): clinical, MRI, operative, histopathology, and follow-up A) Preoperative photograph showing a violaceous, soft, compressible inframammary lesion; B) MRI demonstrating a well-circumscribed, lobulated subcutaneous mass without arterial flow voids or muscle/bone invasion, consistent with a low-flow venous malformation; C) Intraoperative view after subcutaneous dissection and en bloc excision; recipient bed prior to graft placement; D) Immediate postoperative view after inset of a split-thickness skin graft harvested from the thigh; E) Low-power photomicrograph showing dilated vascular cavities within the subcutaneous tissue lined by flattened endothelium; overlying epidermis remains intact (H&E, ×40); F) High-power photomicrograph showing thin-walled venous channels with bland endothelium and fibrous stroma, without atypia (H&E, ×200); G) Six-month follow-up demonstrating stable graft take with a maturing scar.

An otherwise healthy eight-year-old girl was referred to our department for evaluation of a congenital right inframammary mass measuring 57 × 53 mm (Figure 1A). The lesion had been present since birth, enlarged proportionally with growth, and caused intermittent pain, local tenderness, and a progressively distressing contour deformity. In infancy, the mass was misclassified as an infantile hemangioma (IH); oral propranolol was prescribed and monitored in pediatrics per local protocol for approximately seven years, without clinical improvement and without documented cardiovascular adverse events.

On examination, the mass was violaceous, soft, compressible, and non-pulsatile, with no audible bruit or thrill. The overlying skin was intact, without ulceration (Figure 1A). At seven years of age, magnetic resonance imaging (MRI) demonstrated a well-circumscribed, lobulated subcutaneous lesion within the superficial soft tissues that was hyperintense on T2-weighted and iso- to hypointense on T1-weighted sequences, with no arterial flow voids and no invasion of muscle or bone; features consistent with a low-flow venous malformation (VM) (Figure 1B).

Given the persistence of symptoms (pain, tenderness, aesthetic concern) and the absence of response to medical therapy, surgical excision was performed under general anesthesia through a curvilinear inframammary incision. The lesion was carefully dissected from the surrounding adipose tissue and removed en bloc. The defect was reconstructed with a split-thickness skin graft harvested from the thigh (Figure 1C-D).

Histopathological analysis showed dilated, thin-walled vascular channels lined by flattened, bland endothelium without proliferation or atypia, confirming a venous malformation (VM), formerly termed "cavernous hemangioma", an outdated designation (Figure 1E-F). Postoperative recovery was uneventful, and the patient was discharged on postoperative day five. At six-month follow-up, the graft had healed well, with a satisfactory cosmetic result and no evidence of recurrence; the family reported high satisfaction (Figure 1G).

Case two

**Figure 2:**
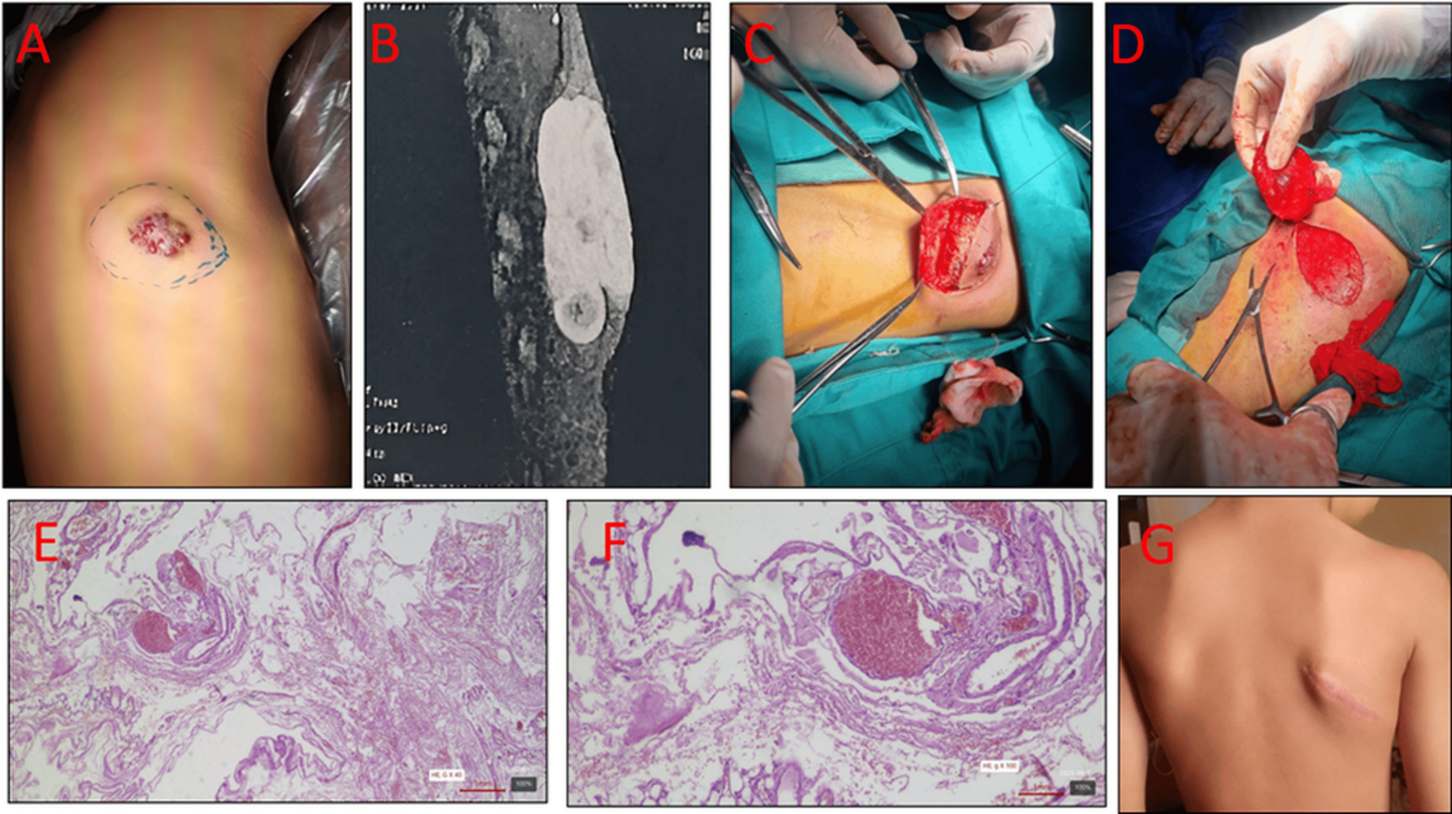
Right dorsal venous malformation (case two): clinical, MRI, operative, histopathology, and follow-up A) Preoperative photograph showing a bluish, soft, compressible mass over the right dorsal region; B) MRI demonstrating a hyperintense lesion on T2 and iso-/hypointense on T1 sequences, without arterial flow voids or muscle invasion, consistent with a low-flow venous malformation; C) Intraoperative field after subcutaneous dissection; recipient bed following complete en bloc excision; D) Excised specimen (en bloc) before hemostasis and wound closure; (E–F) Histopathology showing large, dilated, thin-walled venous channels lined by flattened, bland endothelium with areas of organizing thrombus; no proliferation or atypia, confirming venous malformation (H&E, original magnification ×40; inset ×200).; G) Three-month follow-up demonstrating a fine linear dorsal scar with a satisfactory cosmetic result.

A 10-year-old boy presented to our department for evaluation of a congenital dorsal mass measuring 51 × 23 mm (Figure 2A). The lesion had been present since birth and progressively enlarged with growth. It caused discomfort during prolonged sitting and localized back pain with physical activity; there was no history of bleeding or infection. In infancy, he had received several months of oral propranolol for a presumed infantile hemangioma (IH), without improvement.

On examination, the lesion appeared bluish, soft, compressible, and non-pulsatile, with no audible bruit (Figure 2A). Neurological and musculoskeletal examinations were unremarkable. Magnetic resonance imaging (MRI) demonstrated a well-defined, lobulated subcutaneous lesion overlying the dorsal muscles, hyperintense on T2 and iso- to hypointense on T1 sequences, without muscle invasion or intraspinal extension findings consistent with a low-flow venous malformation (VM) (Figure 2B).

Given the persistence of pain, functional discomfort, and cosmetic impact, surgical excision was performed under general anesthesia through a longitudinal dorsal incision. The lesion was carefully dissected free from surrounding soft tissue with meticulous hemostasis (bipolar cautery) and removed en bloc; the wound was closed primarily in layers (Figure 2C-D).

Histopathological examination revealed large, dilated vascular spaces lined by a single layer of bland endothelium without proliferation or atypia, confirming the diagnosis of VM (Figure 2E-F). The postoperative course was uneventful, and the patient was discharged on postoperative day two. At three-month follow-up, the wound had healed with a fine linear scar and no evidence of recurrence (Figure 2G).

## Discussion

Our cases highlight a persistent diagnostic pitfall: congenital, bluish, compressible subcutaneous lesions that enlarge proportionally with growth are often mislabeled as infantile hemangiomas (IHs) and unnecessarily exposed to β-blockers, despite lacking proliferative biology. Within the ISSVA classification, IHs (vascular tumors) must be distinguished from venous malformations (VMs; vascular malformations), as their natural history and treatment differ fundamentally. Failure to apply this nosology can delay definitive care and subject children to ineffective therapy [1-4].

Clinical examination is central to differentiating IHs from VMs. IHs typically present within the first weeks of life, exhibit rapid proliferation, feel firm or rubbery, and may show warmth or bruit. In contrast, VMs are present at birth, soft, compressible, non-pulsatile, bluish lesions that enlarge proportionally with growth and may cause pain, swelling, or functional impairment [2,5,7]. In both of our patients, compressibility and lack of response to propranolol should have raised early suspicion for VM [3,4].

Imaging plays a pivotal role in avoiding misclassification. Doppler ultrasound, often the first-line modality, can demonstrate venous flow and help exclude high-flow arteriovenous malformations [5-7]. MRI provides decisive features for VMs: T2 hyperintensity, T1 iso-/hypointensity, lobulated contours, absence of arterial flow voids, and occasional phleboliths while also mapping lesion depth and extent for procedural planning [5-7]. Awareness of potential pitfalls (e.g., thrombosis, post-treatment changes, overlap with other low-flow malformations) further reduces diagnostic error and underscores the importance of dynamic acquisitions when indicated [6]. In our patients, MRI findings were prototypical for low-flow VM and directly supported the surgical management strategy.

Histopathology remains essential for definitive diagnosis. Venous malformations are characterized by dilated, thin-walled venous channels lined by flattened, bland endothelium without evidence of proliferation or atypia; intralesional thrombi or phleboliths may also be observed. These findings contrast with infantile hemangiomas, which demonstrate proliferating capillary lobules with mitotically active endothelium. This distinction is clinically significant, as it excludes β-blocker therapy and directs management toward sclerotherapy or surgical excision, while also informing counseling on persistence and recurrence risk [5,7]. In both of our patients, histology confirmed VM and prevented further misclassification.

Management should be individualized within a multidisciplinary team including dermatology, interventional radiology, surgery, and anesthesia. Available therapeutic options include sclerotherapy, surgical excision (primary or staged following embolization or sclerotherapy), thermal ablation, and, in selected diffuse or syndromic cases, targeted medical therapy [2,5,7,8]. For well-circumscribed, superficial VMs associated with pain, functional limitation, or psychosocial distress, complete excision can achieve durable symptom control and favorable cosmetic outcomes when meticulous dissection and hemostasis are performed. Sclerotherapy remains valuable for deeper, diffuse, or poorly demarcated lesions, and as an adjunct to reduce bleeding risk or facilitate staged resection [5,8]. The uncomplicated recoveries and early cosmetic satisfaction observed in our patients are consistent with these reported outcomes [5,8].

Most sporadic VMs harbor somatic variants in TEK (TIE2) and/or PIK3CA, converging on the PI3K-AKT-mTOR signaling pathway [2]. These insights support pathway-directed strategies for complex disease. In patients with PIK3CA-related overgrowth spectrum (PROS) requiring systemic therapy, alpelisib (a PI3Kα inhibitor) has regulatory approval, and accumulating clinical experience supports its role under specialist supervision [9,10]. Such targeted agents are not indicated for small, localized subcutaneous VMs such as ours, where surgery offers an excellent benefit-to-risk balance [5,8].

Limitations of our series include short follow-up (six weeks and one month), which precludes reliable estimates of recurrence, and the small sample size inherent to a case series. Nevertheless, four practice points emerge: Avoid prolonged propranolol in a non-responding "hemangioma"; reassess the diagnosis, confirm low-flow physiology with MRI before intervention, select sclerotherapy versus surgery based on lesion extent, depth, and family goals, and reserve targeted systemic therapy for diffuse or complicated disease after multidisciplinary review [1,3,5,7-10].

## Conclusions

Pediatric subcutaneous venous malformations (VMs) are frequently misdiagnosed as infantile hemangiomas (IHs), leading to ineffective β-blocker therapy and delays in definitive care. Careful bedside assessment identifying soft, compressible, non-pulsatile, bluish lesions present at birth, combined with MRI and confirmatory histopathology, allows accurate diagnosis within the ISSVA framework. When feasible, complete surgical excision provides a safe and definitive treatment, achieving both functional relief and satisfactory cosmetic outcomes with a low risk of recurrence. Strict adherence to standardized classification and imaging pathways prevents unnecessary medical therapy and ensures timely, patient-centered management.

## References

[1] Goldenberg DC, Vikkula M, Penington A, Blei F, Kool LS, Wassef M, Frieden IJ (2025). Updated classification of vascular anomalies. A living document from the International Society for the Study of Vascular Anomalies classification group. J Vasc Anom (Phila).

[2] Kunimoto K, Yamamoto Y, Jinnin M (2022). ISSVA classification of vascular anomalies and molecular biology. Int J Mol Sci.

[3] Léauté-Labrèze C, Hoeger P, Mazereeuw-Hautier J (2015). A randomized, controlled trial of oral propranolol in infantile hemangioma. N Engl J Med.

[4] Léauté-Labrèze C, Dumas de la Roque E, Hubiche T, Boralevi F, Thambo JB, Taïeb A (2008). Propranolol for severe hemangiomas of infancy. N Engl J Med.

[5] Behravesh S, Yakes W, Gupta N, Naidu S, Chong BW, Khademhosseini A, Oklu R (2016). Venous malformations: clinical diagnosis and treatment. Cardiovasc Diagn Ther.

[6] Olivieri B, White CL, Restrepo R, McKeon B, Karakas SP, Lee EY (2016). Low-flow vascular malformation pitfalls: from clinical examination to practical imaging evaluation - part 2, venous malformation mimickers. AJR Am J Roentgenol.

[7] Mulligan PR, Prajapati HJ, Martin LG, Patel TH (2014). Vascular anomalies: classification, imaging characteristics and implications for interventional radiology treatment approaches. Br J Radiol.

[8] Acord M, Srinivasan A (2021). Management of venous malformations. Semin Intervent Radiol.

[9] Singh S, Bradford D, Li X (2024). FDA approval summary: Alpelisib for PIK3CA-related overgrowth spectrum. Clin Cancer Res.

[10] Canaud G, Lopez Gutierrez JC, Irvine AD (2023). Alpelisib for treatment of patients with PIK3CA-related overgrowth spectrum (PROS). Genet Med.

